# Downstream bioprocessing of human pluripotent stem cell‐derived therapeutics

**DOI:** 10.1002/elsc.202100042

**Published:** 2021-09-01

**Authors:** Sebastien Sart, Chang Liu, Eric Z. Zeng, Chunhui Xu, Yan Li

**Affiliations:** ^1^ Laboratory of Physical Microfluidics and Bioengineering Department of Genome and Genetics Institut Pasteur Paris France; ^2^ Department of Chemical and Biomedical Engineering FAMU‐FSU College of Engineering Florida State University Tallahassee FL USA; ^3^ Department of Pediatrics Emory University School of Medicine and Children's Healthcare of Atlanta Atlanta GA USA

**Keywords:** bioprocessing, downstream, pluripotent stem cell, separation

## Abstract

With the advancement in lineage‐specific differentiation from human pluripotent stem cells (hPSCs), downstream cell separation has now become a critical step to produce hPSC‐derived products. Since differentiation procedures usually result in a heterogeneous cell population, cell separation needs to be performed either to enrich the desired cell population or remove the undesired cell population. This article summarizes recent advances in separation processes for hPSC‐derived cells, including the standard separation technologies, such as magnetic‐activated cell sorting, as well as the novel separation strategies, such as those based on adhesion strength and metabolic flux. Specifically, the downstream bioprocessing flow and the identification of surface markers for various cell lineages are discussed. While challenges remain for large‐scale downstream bioprocessing of hPSC‐derived cells, the rational quality‐by‐design approach should be implemented to enhance the understanding of the relationship between process and the product and to ensure the safety of the produced cells.

**ABBREVIATIONS**
AADAC: arylacetamide deacetylase
AcLDL: acetylated low‐density lipoproteins
ALCAM: activated leukocyte cell adhesion molecule
Bim: Bcl‐2 interacting mediator of cell death
Cas9: CRISPR‐associated protein 9
CD: cluster of differentiation
CLRN3: clarin 3
CNTN2: contactin 2
CRISPR: clustered regularly interspaced short palindromic repeats
cTnT: troponin T
CXCR4: C‐X‐C motif chemokine receptor 4
DLX2: distal‐less homeobox 2
EB: embryoid body
ESC: embryonic stem cells
FACS: fluorescence‐activated cell sorting
FOXA2: forkhead box A2
GABA: gamma‐aminobutyric acid
GLUT2: glucose transporter 2
HPA: hepatocyte purifying agent
hPSCs: human pluripotent stem cells
ICG: indocyanine green
iPSCs: induced pluripotent stem cells
KDR: kinase insert domain receptor
LMX1A: LIM homeobox transcription factor 1‐A
LRTM1: leucine‐rich repeats and transmembrane domains 1
MACS: magnetic‐activated cell sorting
MBs: molecular beacons
miRNA: microRNA
MLC: myosin regulatory light chain
MYF5: myogenic factor 5
MYH7: myosin heavy chain beta
NCAM: neural cell adhesion molecule
NG2: neural/glial antigen 2
NKX2‐5: NK2 homeobox 5
NPCs: neural progenitor cells
NPPA: natriuretic peptide A
NURR1: nuclear receptor related 1 protein
Oct‐4: octamer‐binding transcription factor 4
OPCs: oligodendrocyte progenitor cells
OSR: odd‐skipped related transcription factor
PAX7: paired box 7
PDGFRα: platelet‐derived growth factor receptor α
PDX‐1: pancreatic and duodenal homeobox 1
PE: pancreatic endoderm
PNIPAAm: poly(*N*‐isopropylacrylamide)
QbD: quality by design
ROCK: rho‐associated protein kinase
RPE: retinal epithelial cells
SIRPA: signal–regulatory protein alpha
SIX2: SIX homeobox 2
SLC10A1: solute carrier family 10 member 1
SMAD: small mothers against decapentaplegic
SOX: sex determining region Y‐box
SSEA: stage‐specific embryonic antigen
TALEN: transcription activator‐like effector nucleases
TBX5: T‐Box transcription factor 5
TH: tyrosine hydroxylase
TH: tyrosine hydroxylase
Tra: tumor‐related antigen

## 1 INTRODUCTION

Human pluripotent stem cells (hPSCs) provide an alternative cell source for a variety of somatic tissues due to their unique abilities to self‐renew and to differentiate into nearly all types of cells. hPSC derivatives have been tested in several phase I clinical trials for their potential use as therapeutic products and also evaluated for drug discovery and disease modeling [1, 2, 3]. Initially, the development and manufacturing of hPSC‐derived cells have focused on the optimization of differentiation efficiency, i.e. the upstream bioprocessing, leading to improved differentiation protocols that allow the production of lineage‐specific cells at high efficiency and purity [3, 4, 5, 6, 7]. For examples, oligodendrocyte progenitor cells (OPCs) and neural progenitor cells (NPCs) can be generated at 70–90% purity from hPSCs using either embryoid body (EB)‐based protocol or the monolayer protocol via dual inhibition of SMAD (Small Mothers Against Decapentaplegic) signaling [8, 9], and 30–90% pure cardiomyocytes can be produced using either growth factor‐ or small molecule‐guided protocols [10, 11, 12]. The development of such efficient differentiation protocols demonstrates great progress in the upstream bioprocessing for the production of hPSC‐derived cells [5]. However, downstream bioprocessing (i.e. cell separation), a critical step and the bottle neck to finalize the hPSC‐derived products, requires further investigation [13].

**FIGURE 1:**
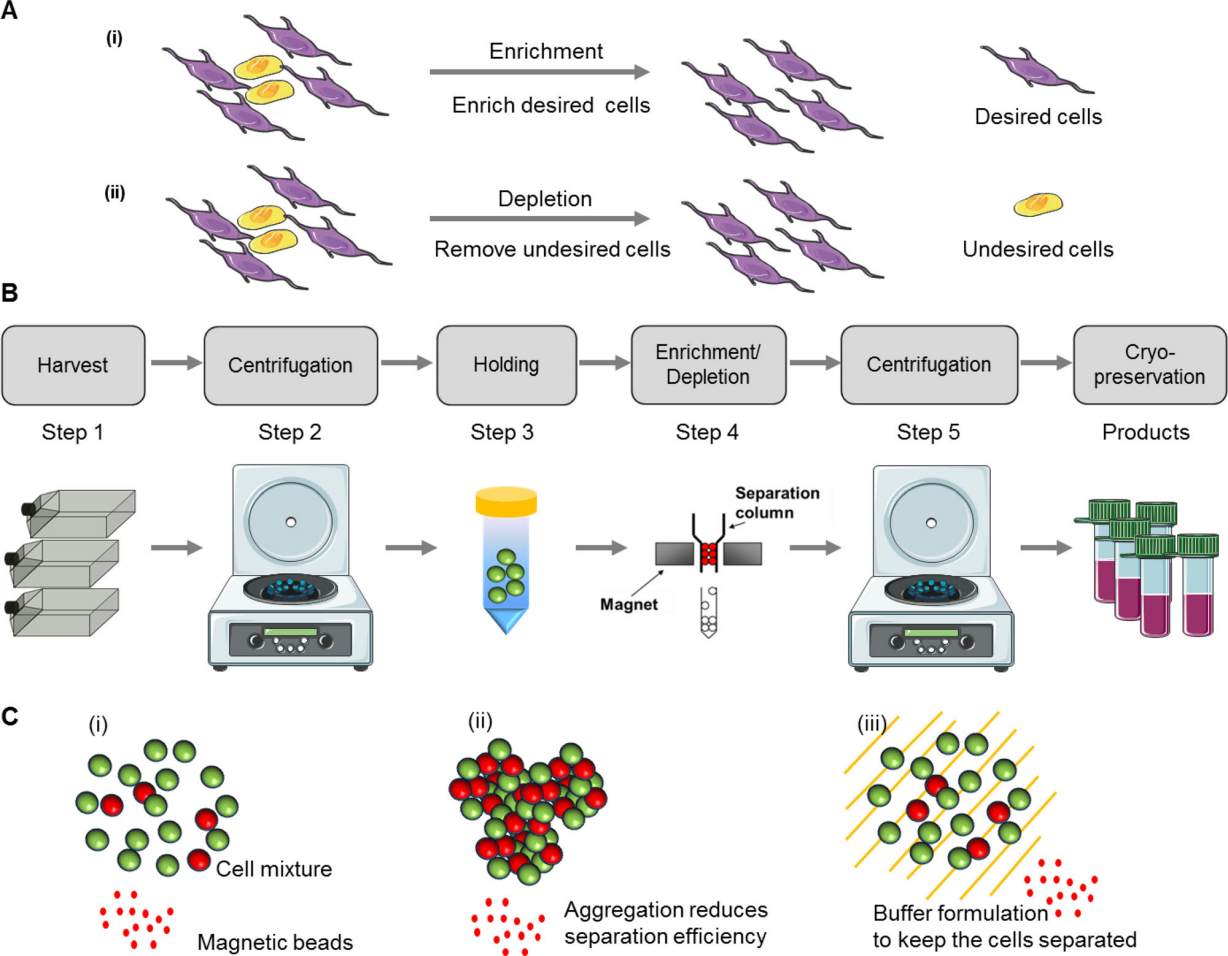
Downstream bioprocessing for hPSC‐derived products. (A) Enrichment and depletion strategies to isolate hPSC‐derived cells. (i) Enrichment: to collect the desired cells (suitable for low differentiation efficiency). (ii) Depletion: to remove undesired cells (suitable for high differentiation efficiency). (B) The major steps of downstream processing. (1) Cell harvesting; (2) centrifugation to remove harvesting enzyme; (3) holding to wait for all the cells to be harvested; (4) depletion to remove the unwanted cells; (5) centrifugation to remove the depletion buffer; and (6) fill and finish to transfer the cells in the cryopreservation buffer for cryopreservation. The overall yield after these steps is expected to be 59% if each step has a 90% yield in an ideal situation. (C) Effects of aggregation on large‐scale cell labeling. (i) A single cell suspension allows cell labeling with magnetic beads. (ii) If aggregation happens, the targeted cells cannot be equally labeled and the unlabeled cells in the aggregates are removed, which reduces the yield. (iii) The cells having high aggregation tendency are kept in the formulated buffer as single cell suspension to improve the processing efficiency. This figure contains images from Servier Medical Art (smart.servier.com)

Since current differentiation processes from hPSCs usually result in a mixture of cell types including residual undifferentiated cells [14], downstream bioprocessing needs to be in place for selective purification of desired cell population or removal of unwanted cell populations (Figure 1A). For example, a final stem cell product without undifferentiated cells or progenitors of undesired lineages is critical in order to reduce the risk of teratoma formation after transplantation [14]. Here, we review the emerging downstream bioprocessing for hPSC‐derivatives, including recent advances in cell separation after hPSC expansion and differentiation. We provide examples of the identification of novel lineage‐specific surface markers, which can be targeted for the separation of hPSC‐derived cells. In addition, we discuss methods and practical process flow for hPSC downstream bioprocessing and specific challenges facing the production of neural and cardiac cells.

## 2 METHODS TO PURIFY HPSC‐DERIVED PRODUCTS

During differentiation, hPSCs and derivatives possess stage‐specific properties in cell density, expression of surface markers, metabolic requirement, and adhesion strength. Based on these and other characteristics, different types of separation processes for stem cell‐derived cells have been evaluated [14], including standard separation technologies such as density‐based separation and fluorescence‐activated cell sorting (FACS) and magnetic‐activated cell sorting (MACS) based on specific surface markers. Novel separation strategies, such as cell separation based on differential adherence to culture vessels [15, 16, 17] and selective removal of cells based on distinct metabolic activity [18, 19], have also been developed with the better understanding of hPSC properties.

### 2.1 Density‐based separation

A mixture of cell types with different cell densities could be produced in differentiation cultures and may be separated according to their densities. One example is Percoll gradient centrifugation to enrich hPSC‐derived cardiomyocytes [20]. Cells harvested from the differentiation culture are loaded onto the two layers of Percoll and then centrifuged. Majority of the cardiomyocytes will be present in the lower layer of Percoll, and non‐cardiomyocytes will be in other fractions. Up to 95% pure cardiomyocytes can be obtained from a starting differentiation culture containing about 50% cardiomyocytes [11]. This method, however, has low resolution and is not amenable for large scale separation.

### 2.2 Separation based on cellular biophysical properties

Substantial differences in biophysical properties among undifferentiated hPSCs, partially reprogrammed cells, somatic cells, and hPSC‐derived differentiated cells have been observed [21]. For example, adhesive properties [15], plasmic membrane rigidity [22], and optical characteristics [23] have been shown different at various developmental stages of hPSCs. The changes in these properties can be used as targets for the selection of specific committed populations.

hPSC‐derived cardiomyocytes display specific second harmonic signal from myosin rod bundles, which enables their separation form an heterogeneous population of differentiated cells [23]. Alternatively, neural crest cells derived from the replated differentiating hESC aggregates were spontaneously segregated from the main cellular mass, which enable their isolation by shape selection and manual picking [24].

On the other hand, expanded pigmented cells derived from mouse induced pluripotent stem cells (miPSCs) were demonstrated to be more adhesive than non‐pigmented cells [15]. This property enables their purification by regulating the time of exposure and the type of enzymatic detachment: non‐pigmented colonies can be recovered by a 5‐min treatment with Accutase and pipetting, while the remaining adhesive pigmented cells can be secondarily recovered with trypsin [15]. Similarly, medium supplementation with ROCK (Rho‐associated protein kinase) inhibitor (Y‐27632) prior to cell dissociation, enables the removal of less adhesive cells and thus, to enrich the population with endothelial progenitor cells [16]. Moreover, the exit from pluripotency was found to increase cell membrane rigidity. These changes in membrane fluidity and lipid composition can be linked to the differentiation stage. Based on this principle, cell separation can be performed for selecting various differentiated cells as the function of their adhesive properties under membrane fluidization conditions [22].

Human induced pluripotent stem cell (hiPSC) colonies can be detached at a shear stress of 85–125 dynes/cm^2^ within 4 min of fluid‐flow application, while the differentiated cells remain attached due to higher adhesive strength. This method resulted in the isolation of fully reprogrammed iPSC colonies to >95% purity from heterogeneous reprogrammed cultures and differentiated progenies. However, this method has not been evaluated for lineage‐specific differentiated cells.

Because cell/adhesive surface interactions are regulated upon differentiation, this has led to the development of novel biomaterials for the cell isolation of particular differentiated phenotype [25, 26]. At the undifferentiated stage, iPSC colonies can be selectively detached from thermosensitive polymer (PNIPAAm (poly(*N*‐isopropylacrylamide)), at low temperature (i.e. 22°C), while committed cells remain attached on the surface [25]. This method enables a non‐invasive enrichment of undifferentiated stem cell population. Similarly, hiPSCs committed to cardiac lineages seeded on poly(*N*‐isopropylacrylamide) coated with laminin‐521, which has a lower critical solution temperature at 8°C, promotes the specific detachment of cardiomyocytes [27]. In addition, it was found that committed cells can be eliminated by a high‐speed laser irradiating specific areas of light‐responsive polymers (i.e. poly[(methylmethacrylate)‐co‐(Disperse Yellow 7 methacrylate)] layers) [26]. Alternatively, laminins of different isoforms promote different adhesion strength of various differentiated cell types. For instance, LN211/332/511E8 promotes the adhesion of non‐epithelial, while LN332/511E8 favor the attachment and the proliferation of epithelial cells [17]. The specificity of the cell binding to different laminin isoforms has enabled the purification of corneal epithelium cells from heterogeneous differentiated cells [17].

### 2.3 Selective cell removal based on metabolic activity

Based on the marked biochemical differences in glucose and lactate metabolism between cardiomyocytes and non‐cardiomyocytes, high purity (up to 99%) of cardiomyocytes can be obtained from hPSC differentiation culture [18]. The undifferentiated hPSCs and non‐cardiac cells that mainly depend on glycolysis are not able to survive under glucose‐depleted and lactate‐abundant conditions, whereas cardiomyocytes can survive by using lactate as an alternative energy source. Therefore, cardiomyocytes preferentially survive in glucose‐depleted culture medium supplemented with lactate and are consequently enriched. Similarly, using medium depleted with glucose and supplemented with fatty acid and 3,3′,5‐triiodo‐l‐thyronine (i.e. a molecule that promotes fatty acid oxidation and mitochondria biogenesis), the selection and the maturation of cardiomyocytes were reported [19].

Retinal epithelial cells (RPE) were found to express high levels of lipoprotein receptors internalize AcLDL (Acetylated Low‐Density Lipoproteins). Based on this principle, Dil conjugated‐AcLDL was used for the specific labelling and the enrichment of differentiated RPE population [28]. As another example, indocyanine green (ICG) is specifically internalized by hepatocytes. By modifying the fluorescent emission properties of ICG, a hepatocyte purifying agent (HPA, *λ*_em_  =  562 nm) has been designed for the labelling and in vitro sorting of purified hepatocytes derived from hPSCs [29].

### 2.4 Negative selection

Contrary to positive cell selection, other methods have been developed for the removal of undesired cell types by specifically inducing their apoptosis, thus improving the recovery yield of desired phenotypes. For instance, the transfection of PSCs with the synthetic microRNA (miRNA) switches (i.e. that reduces the translation level of an associated protein in the absence of target miRNA), miR‐Bim (Bcl‐2 interacting mediator of cell death)‐switch, induces the selective apoptosis in undifferentiated cells while maintaining the differentiated cardiomyocytes [30]. Alternatively, the lectin rBC2LCN‐PE23 was found to selectively bind and internalize specifically to undifferentiated stem cells and to induce their apoptosis [31]. This compound was found to efficiently reduce the risk of teratoma formation.

### 2.5 Separation by FACS

The FACS equipment can detect fluorescence signals from cells labeled with fluorochrome‐coupled antibodies. Based on the characteristics of labeling and the targeted markers (usually surface antigens), FACS allows sorting and separation of individual cells into different populations (e.g. SSEA (Stage‐Specific Embryonic Antigen)‐4 positive or SSEA‐4 negative). An advantage of FACS is that it allows serial and multi‐parametric separation based on multiple surface markers with high selectivity, although cell viability may be affected by the sorting procedure [32]. However, the throughput of FACS is limited with 5 × 10^3^ to 7 × 10^4^ cells sorted per second. Therefore, FACS is more suitable for small‐scale cell separation in research use.

### 2.6 Separation by MACS

MACS is a technique similar to FACS but uses magnetic particles that carry antibodies targeting specific cell surface antigens. In a magnetic field, the magnetically labelled cells will be retained in the column and the unlabeled cells will flow out. For example, cardiomyocytes were enriched to 95% from hPSC differentiation culture by MACS that targeted the cardiomyocyte‐associated surface marker VCAM1 (Vascular Cell Adhesion Molecule 1) [33]. However, while MACS is an appealing approach to selectively remove unwanted cells from a cell mixture, it has its limitations. For example, a modeling study suggests that an impractical number of repetitive MACS would be needed to achieve the clearance of undifferentiated stem cells positive for SSEA‐1 from a pool of differentiated and undifferentiated cells [34]. However, the affinity of the magnetic beads could also be improved with novel surface modifications to improve sorting efficiency [35]. An advantage of MACS is that it is more practical than FACS for large‐scale processing due to the lower cost and the commercial availability of automated, closed‐systems [36]. An alternative of MACS beads, the SpheriTECH, enables the purification of photoreceptor progenitor cells derived from hiPSCs without purification label, by simply sorting cells through affinity binding on large beads [37].

### 2.7 Separation using microfluidics

Microfluidics has recently been demonstrated as efficient tools for hPSC culture, characterization and screening [38]. Microfluidics can also serve for the separation of heterogeneous population of differentiated cells. For instance, microfluidics rolling columns have been fabricated for the selective isolation of SSEA‐1^+^ positive cells [39]. The boundaries of the rolling column chips were coated with an antibody that reduced the rolling velocity of the SSEA‐1^+^ cells, while committed cells flow out of the channel [39]. Consequently, SSEA‐1^+^ cells are retained for longer time within the channel and can consequently be separated from the differentiates cells. Similar operations were performed by covering ridges patterning the floor of microfluidic chips with an anti‐Tra (Tumor‐related antigen)‐1‐60 antibody, which eliminates undifferentiated cells from cardiomyocytes derived from hiPSCs [40].

## 3 SURFACE MARKER IDENTIFICATION FOR THE ISOLATION OF HPSC‐DERIVED CELLS

Since FACS and MACS rely on the detection of surface markers, it is important to identify specific surface markers of different lineages during different stages of hPSC differentiation. We discuss examples of studies on surface markers for undifferentiated cells and derivatives of three germ layers including neural cells, cardiomyocytes, and pancreatic progenitors (Table 1).

**TABLE 1:** Methods used to isolate undifferentiated hPSCs or hPSC‐derived cells

Isolated cell type	Methods	Separation basis	Separation performance	Reference
Undifferentiated hPSCs	FACS and MACS	SSEA‐4, Tra‐1‐60/81	FACS: all the pluripotent cells removed, lower viability; MACS: 81–84% removed, higher viability	Fong et al., 2009 [41]
	FACS	SSEA‐5 (with CD9/CD90 or CD50/CD200)	Teratoma‐forming cells completely removed	Tang et al., 2011 [43]
	Flow shear	Adhesion strength	Undifferentiated hPSCs enriched to 95–99%; fast separation (10 min)	Singh et al., 2013 [21]
	FACS and MACS	Lectin	Pluripotent cells removed from mixed populations	Wang et al., 2011 [45]
	Light response polymer	Irradiation of differentiated cells	Purity >98%: TRA‐1‐60	Hayashi, et al. 2018 [26]
	Lectin specific binding on undifferentiated cells	Lectin fusion with a toxin induce apoptosis	Remaining undifferentiated cells <0.1%	Tateno et al., 2015 [31]
hPSC‐derived neural progenitors	FACS and MACS	FORSE‐1 NCAM (CD56)	98% purity for FORSE‐1; The isolated NCAM^+^ cells had neuronal morphology and express nestin and β‐tubulin III	Pruszak et al., 2007 [32]
	FACS	CD184^+^/CD271^−^/CD44^−^/CD24^+^	Selection for neural stem cells; CD184^−^/CD44^−^/CD15^low^/CD24^+^ for neurons; CD184^+^/CD44^+^ for glial cells; >90% purity	Yuan et al., 2011 [46]
	FACS	CD15, CD24, CD29	CD15^+^/CD29^HI^/CD24^LO^ defined neural stem cells; CD15^−^/CD29^LO^/CD24^HI^ selected neuroblasts and neurons; >95% purity	Pruszak et al., 2009 [51]
	FACS	CORIN	Midbrain dopaminergic progenitors isolated and further differentiated into dopaminergic neurons in vivo.	Doi et al., 2014 [52]
	MACS	CD271, CD133	Purity >80%	Bowles et al., 2019 [47]
	FACS	PSA‐NCAM, CNTN2	Purity >80%	Fathi et al., 2019 [53]
	FACS	LRTM1	Purity 30–40%: TH, FOXA2 and NURR1	Samata et al., 2016 [55]
hPSC‐derived cardiomyocyte progenitors	FACS	KDR^low^/C‐kit^neg^	Cardiac progenitors isolated and differentiated to cardiomyocytes with >50% purity	Yang et al., 2008 [59]
	FACS	KDR^+^/PDGFR‐α^+^	Cardiac progenitors isolated and differentiated to cardiomyocytes with >80% purity	Kattman et al., 2011 [60]
hPSC‐derived cardiomyocytes	MACS	VCAM1^+^	95% of cells expressing cardiac troponin T isolated	Uosaki et al., 2011 [33]
	MACS	ALCAM1^+^	60% of cardiomyocytes isolated	Rust et al., 2009 [62]
	FACS	SIRPA^+^	Up to 98% cardiomyocytes from sources comprising 40–50% cardiomyocytes isolated	Dubois et al., 2011 [61]
	Fed with glucose‐depleted lactate‐abundant medium	Distinct metabolic flow for different cell types	Up to 99% pure cardiomyocytes obtained; the preparation did not form tumors after transplantation	Tohyama et al., 2013 [18]
	Percoll gradient centrifugation	Cell density	70‐95% cardiomyocytes isolated	Xu et al., 2002 [11, 20]
	Thermoresponsive polymer	Adhesion strength	Purity >90%: TnnT	Sung et al., 2019 [27]
	Glucose‐depleted medium with fatty acid and 3,3′,5‐triiodo‐l‐thyronine	Distinct metabolic flow for different cell types	Purity >95% cardiac troponin T	Lin et al. 2017 [19]
	miR‐Bim‐switch	Distinct miRNA expression for different cell types	Purity >90%: TnnT	Miki et al., 2015 [30]
hPSC‐derived pancreatic progenitors	FACS and MACS	CD142 CD200 and CD318	Pancreatic endoderm (CD142) and endocrine cells separated; FACS: ‐95% CD142^+^ cells; MACS: 75–90% CD142^+^ cells	Kelly et al., 2011 [65]
	FACS	CD133^+^/CD45^−^/CD34^−^	99% purity obtained that had multi‐lineage differentiation capacity	Oshima et al., 2007 [70]
	FACS and MACS	GLUT2	FACS: higher purity, lower yield; MACS: lower purity, higher yield.	Segev et al., 2012 [66]
	FACS	CD24^+^NCAM^−^	Purity to >70%, PDX‐1^+^ progenitors enriched	Jiang et al., 2011 [67]
miPSC‐derived retinal pigmented cells	Enzymatic treatment	Adhesion strength	Purity >98%	Iwasaki et al., 2016 [15]
hiPSC‐derived retinal pigmented cells	Dil conjugated‐AcLDL uptake	Specific molecule uptake	Pure functional RPE monolayer	Michelet et al., 2020 [28]
hiPSC‐derived endothelial progenitor cells	Enzymatic treatment; with Y‐27632	Adhesion strength	Purity >90%: PECAM1, CDH5, and CD34	Aoki et al., 2020 [16]
hiPSC‐derived corneal epithelial cells	Adhesion on different laminin isoform	Adhesion strength	Purity ∼85%: CD200^−^/SSEA‐4^+^	Shibata et al., 2020 [17]
hPSC‐derived hepatocytes	Hepatocyte purifying agent uptake	Specific molecule uptake	Purity >90%: albumin	Park et al., 2019 [29]
hiPSC‐derived renal progenitor cells	FACS	CD9^−^/CD140a^+^/CD140b^+^/CD271^+^	Purity >70%: OSR1, SIX2 and HOXD11	Hoshina et al., 2018 [73]

### 3.1 Surface markers for undifferentiated cells

Surface markers such as Tra‐1‐60, Tra‐1‐81, and SSEA‐4 have been used to identify and remove undifferentiated hPSCs (SSEA‐1 for mouse embryonic stem cells, mESCs) [41, 42]. For example, FACS and MACS can separate SSEA‐4 and Tra‐1‐81 labeled hESCs (human embryonic stem cells) from other cells [41], and FACS targeting SSEA‐5, CD (Cluster of Differentiation)9 and CD90, markers of pluripotent stem cells, can remove cells with teratoma‐formation potential from incompletely differentiated hESC cultures [43]. Podocalyxin‐like protein‐1 is also highly expressed in undifferentiated cells, and a cytotoxic antibody recognizing podocalyxin‐like protein‐1 has been used to selectively kill undifferentiated hESCs [44]. In addition, specific glycosylation of surface proteins can distinguish pluripotent cells from non‐pluripotent cells; therefore, lectins with distinctive binding ability to carbohydrates have been used to remove hPSCs [45].

It should be noted that teratomas could also originate from the precursors that still have stem cell features and are not completely differentiated; thus, these precursors also need to be removed from stem cell‐derived products. In any case, for safe cell therapy, it is critical to determine depletion criteria and assay sensitivity of assays to detect undifferentiated stem cells or precursors.

### 3.2 Surface markers for neural differentiation

Current neural differentiation methods for hPSCs result in cellular heterogeneity with respect to developmental stages and lineage specifications. CD133, A2B5, CD29, CD146, NCAM (Neural Cell Adhesion Molecule) (or CD56) and CD271 were reported to be surface markers on neural progenitor cells (NPCs), and CD24 and NCAM are surface markers for neurons. Targeting CD24 or NCAM by FACS enabled the isolation of differentiated neurons [32]. Isolation of NPCs, neurons, and glia cells can also be achieved with combinations of markers (e.g. CD184^+^/CD271^−^/CD44^−^/CD24^+^ for NPCs; CD271^−^/CD133^+^ for neurons) [46, 47], and different combinations can be used to delineate NPCs: CD184^+^/CD326^−^ [48], CD133^+^/CD45^−^/CD34^−^ [49], the expression of CD133, CD15, and GCTM‐2 [50], and the expression of CD24, CD15, and CD29 [51]. Recently, a floor plate marker CORIN has been used to isolate human iPSC‐derived dopaminergic progenitors [52]. Using a LMX1A^EGFP^ (LIM homeobox transcription factor 1‐A) reporter cell line, novel membrane markers that can be selectively enriched in dopaminergic neurons derived from hESCs have been identified, such as polysialylated embryonic form of neural cell adhesion molecule (PSA‐NCAM) and contactin 2 (CNTN2) [53]. Other studies have identified novel surface markers to positively enrich midbrain dopaminergic neurons such as LRTM1 (Leucine‐Rich repeats and TransMembrane domains 1), CORIN and CD166, or markers to deplete the undesired cells (CXCR4, C‐X‐C Motif Chemokine Receptor 4) after the sorting of LMX1^+^ FOXA2 (Forkhead Box A2)^+^ cells [54, 55].

Surface markers reported for oligodendrocyte progenitors include NG2 (Neural/glial antigen 2), PDGFRα (Platelet‐Derived Growth Factor Receptor α), and DLX2 (Distal‐Less Homeobox 2) [56] or PDGFRα/CD140 [57]. However, it is difficult to isolate oligodendrocytes based on a single marker because there exists a vast degree of heterogeneity in cellular phenotypes [58].

### 3.3 Surface markers for cardiomyocyte differentiation

Markers have been identified both for cardiac progenitor cells and the mature cardiomyocytes. KDR (Kinase insert Domain Receptor)^low^/C‐KIT^neg^ cardiac progenitor cells express high levels of cardiac transcription factors and can generate >50% cardiomyocytes after further differentiation [59]. Similarly, the KDR^+^/PDGFRα^+^ cardiac progenitor cells can differentiate into >80% cardiomyocytes [60]. In addition, cardiomyocyte‐specific surface markers have also been identified, which include signal–regulatory protein alpha (SIRPA), VCAM1, and activated leukocyte cell adhesion molecule (ALCAM) [33, 61, 62]. Following selection based on SIRPA from starting cultures that were ∼40–50% cardiac troponin T positive (cTnT^+^), 90–98% of the cells were positive for cTnT^+^ [61]. Selection based on VCAM1 by MACS enabled enrichment of cTnT^+^ cells up to 95% [33]. Compared with these positive selection methods, depletion of unwanted cell population may be considered for cultures with high efficiency of cardiomyocyte differentiation (80‐90%). Similarly, cardiomyocytes can be purified using VCAM1‐coupled magnetic Dynabeads [63] or integrins (α1, α5, and α6) and N‐cadherin to more than 90% purity [64].

### 3.4 Surface markers for endoderm, hepatic, renal, and pancreatic cell differentiation

hPSC‐derived pancreatic cells are heterogeneous. CD142 has been identified as a marker for pancreatic endoderm (PE) cells which give rise to islets with glucose‐responsive insulin‐secreting cells, and CD200 and CD318 are known as the markers for endocrine cells [65]. Targeting markers such as CD142 can therefore allow enrichment of PE cells, which has been shown to also reduce the probability of teratoma formation (from 46 to 0%) [65]. GLUT2 (Glucose transporter 2), which is co‐expressed with PDX‐1 (Pancreatic and Duodenal homeobox 1) [66], and CD24, which is shown to correlate with PDX‐1^+^ cells [67], have also been evaluated to enrich pancreatic progenitor cells derived from hPSCs. In addition, surface markers CXCR4, CD49e, CD141 and CD238 have been identified in a study targeting SOX17 to isolate hESC‐derived endoderm cells [68, 69]. Although CD133 has been reported as a marker for pancreatic progenitors in mouse, it may have a broader expression other than in pancreatic PDX‐1 positive cells in humans [70]. More mature endoderm cells such as hepatocytes derived from PSCs can also be isolate by specific membrane markers (e.g. SLC10A1 (Solute Carrier Family 10 Member 1), CLRN3 (Clarin 3), or AADAC (Arylacetamide Deacetylase)) [71], or ASGR1 (i.e. Asialoglycoprotein Receptor 1) [72]. OSR (Odd‐Skipped Related Transcription Factor)1^+^/SIX2 (SIX homeobox 2)^+^ renal progenitor cells have also been isolated by a set of membrane markers, such as CD9^−^/CD140a^+^/CD140b^+^/CD271^+^ cells [73]. Similarly, corneal epithelial cells can be selected by selectively depleting CD200^+^ cells [74].

However, questions remain if the cell populations identified by different marker sets are the same cells at different stages or different cells along similar developmental track. For the production purpose, the isolated population needs to be fully characterized and demonstrate the consistent attributes for the targeted applications.

### 3.5 Non‐surface markers for the separation of hPSC‐derived cells

Markers other than those expressed on cell membrane but specific to particular cells may be leveraged to isolate these cells. For example, mRNA in live cells can be detected by molecular beacons (MBs) which are stem‐loop (hairpin) oligonucleotide probes with a fluorophore at one end and a quencher at the other end [75]. MBs targeting Oct‐4 (Octamer‐binding transcription factor 4) or Sox2 (Sex determining region Y‐box 2) can separate undifferentiated PSCs from the differentiated cells [76, 77]; MBs targeting cardiomyocyte marker myosin heavy chain beta (MYH7) can enrich cardiomyocytes derived from mouse and human embryonic stem cells [78]; and MB targeting NPPA (Natriuretic Peptide A) mRNA, a marker known to be associated with early‐stage working‐type cardiomyocytes, can enrich working‐type cardiomyocyte from cardiomyocyte differentiation culture [79]. For cell separation with MBs, careful design and extensive validation of the MB probes targeting the desired gene are critical.

### 3.6 Knock‐in reporter cell lines

Applications of genome editing technologies (e.g. CRISPR/Cas9 (clustered regularly interspaced short palindromic repeats/CRISPR‐associated protein 9), TALEN (transcription activator‐like effector nucleases) etc.) have enabled the construction of novel cells lines that are rendered fluorescent upon expression of specific markers or purified with a specific phenotype, through induction of antibiotic resistance. For instance, CRISPR/Cas9 editing of hiPSCs has enabled the generation of double reporter TBX5 (T‐Box Transcription Factor 5)^Clover2^ and NKX2‐5 (NK2 Homeobox 5)^TagRFP^ cells that has been used for the purification of heart cell subtypes, such as cells from the first heart field, epicardial, second heart field, and endothelial lineages [80]. Alternatively, NKX2‐5^eGFP/w^ and MLC (Myosin regulatory Light Chain)2v^mCherry/w^ reporter cells have been generated to isolate ventricular like cells [81]. Genome editing (TALEN) have also been used to introduce a selection marker (neomycin) or GFP after the locus of myosin light chain 2 (MYL2) that can serve for identifying and selecting ventricular cardiomyocytes [82]. Similarly, introduction of a Zeocin resistance gene under control of the cardiac‐specific α‐myosin heavy chain (i.e. α‐MHC, MYH6) promoter has been used to purified iPSC‐derived cardiomyocytes (iPSC‐CMs) in a murine model of myocardial infarction [83].

Similar strategies have been used for the identification and the selection of myogenic derivatives. For instance, a CRISPR/Cas9 mediated homologous recombination method has been used to select MYF5 (Myogenic Factor 5)^GFP^ muscle progenitors [84]. The construction of a double reporter PAX7 (Paired box 7)^tdTomato^ and MYF5^EGFP^ enabled the purification of muscles stem cells (satellite cells) [85], while a doxycycline induced PAX7 expression system promoted the generation of homogeneous population of skeletal myogenic progenitors [86].

Reporter cells have also been generated for the purification of neural derivatives. For instance, introduction of a human vesicular GABA (Gamma‐Aminobutyric Acid) transporter (hVGAT) promoter to drive the expression of mCherry has enable the isolation of GABAergic neurons [87], while midbrain dopaminergic neurons can be selected using tyrosine hydroxylase (TH)^RFP^ construction [88]. However, in view of therapeutic applications of hPSC derivatives, such methods may have increased risk of tumorigenicity induced by genome modification [89].

## 4 WORKFLOW OF DOWNSTREAM BIOPROCESSING FOR HPSC‐DERIVED CELLS

Downstream bioprocessing in the production of hPSC‐derived cells involves multiple steps which will accumulatively reduce the yield of the process from each step (Figure 1B) shows an example using MACS depletion). The major steps are: (1) cell harvesting: dissociation of the cells from adherent surface of a large amount of culture vessels; (2) centrifugation or concentration and medium exchange: to remove harvesting enzyme and wash the cells; (3) holding: the harvested cells are held in the buffer to wait for all the cells to be harvested and centrifuged; (4) depletion: to remove the unwanted impure cells; (5) centrifugation: to remove the depletion buffer and resuspend in the cryopreservation buffer; (6) cell formulation for cryopreservation: to transfer the cells in the cryopreservation buffer to the cryovials and get ready for cryopreservation. Due to these many steps, the final yield would be 50–60% if each step has a yield of 90% (ideal situation) and only 30–35% if each step has a yield of 80% (good situation). Therefore, more than half of the cells could be lost due to the downstream processing, which usually happens in one working day after months of cell expansion and differentiation. Hence, understanding the operation parameters for each step is definitely required to minimize the cell loss while maintaining functional viable cells.

### 4.1 Cell harvesting

The critical components of cell harvesting from a large number of vessels are the harvesting enzyme and the washing volume. High concentration of enzyme or inappropriate dissociation solutions or duration could reduce cell viability and/or could not remove all the cells on the surface. For differentiation culture, trypsin has been the common enzyme, but its concentration and incubation time need to be optimized for specific cell types. New non‐enzymatic passaging method using sodium citrate, which is formulated as a hypertonic solution, has been used to dissociate multicellular aggregates of hPSCs [90]. For bioprocessing, reduced harvesting volume is desired, which requires the balance between low volume and cell loss due to insufficient washing. New advancements in improving the hPSC harvesting have been made by using thermoresponsive hydrogels to release hPSCs by changing the temperature from 37°C to 4°C [91].

### 4.2 Centrifugation

The parameters during centrifugation include speed, time, cell suspension volume, and cell concentration. Variations in sedimentation have been observed for different cell collections which trigger multiple centrifugations. The harvesting enzyme, the quenching buffer, and the waiting time may all contribute to the sedimentation performance. For a large number of vessels, development of a large‐scale centrifugation process (such as using bags) is required to reduce the time on centrifugation step.

### 4.3 Holding buffer and holding temperature

Holding process becomes significant when the number of vessels is high (>100 T‐flasks) and the processing time is long (as long as 6–8 h). In order to pool all the cells together for follow‐up processing, the cells harvested first have to sit in the buffer until the harvesting step is completed. Hence, the holding buffer and the holding temperature (37°C, 25°C, or 4°C) need to be evaluated to maintain cell viability. To formulate the holding buffer, one should take consideration of cell aggregation. Although the harvested cells are in single cell suspension, the cells may self‐assemble into aggregates during the holding period. The degree of aggregation depends on cell type, cell concentration, and holding time. The formation of aggregates will affect the efficiency of cell labeling and separation in subsequent steps, significantly reducing cell yield.

### 4.4 Depletion buffer formulation and volume

Depletion is applied to remove the small number of impure cells when the population has a high percentage of desired cells. This process can be done by MACS targeting surface markers on the undesired cells. The requirement for depletion buffer is similar to holding buffer: maintaining cell viability and preventing cell aggregation. The aggregated cells do not allow homogeneous labeling with the antibody‐conjugated beads and easy cell‐bead separation (Figure 1C). Consequently, the depletion buffer needs to be carefully formulated. The cell concentration and the volume for depletion affect the depletion scale. High cell concentration (>10^7^ cells/mL) and low volume (<100 mL) are preferred, while cell loss needs to be minimized. Since multiple markers could be used for depletion, this step may be repeated to remove different cell populations.

### 4.5 Formulation for cryopreservation

Cryopreservation buffer and volume are the important parameters of this step. In general, the cryopreservation buffer depends on the differentiated cell types of interest, and the cryopreserved cell concentration depends on the intended applications. For transplantation studies, high concentration of cells (10^7^‐10^8^ cells per vial) may be required to minimize the time for thawing process and the number of vials to achieve the desired dose of cells.

## 5 CHALLENGES IN DOWNSTREAM PROCESSING HPSC‐DERIVED PRODUCTS

### 5.1 Challenges in downstream processing for hPSC‐derived neural progenitor cells

Large‐scale purification of neurons derived from PSCs has been achieved using MACS, yielding to the recovery of up to 10^5^–10^6^ neurons (more than 90% purity) [92]. However, specific challenges remain for downstream processing of these specific lineages. In the case of neural progenitor cells, the harvesting cell density is relatively low (about 1 × 10^6^ cells per cm^2^), which could require to harvest cells from a large number of vessels. If the higher harvesting density can be reached, the number of required vessels will be significantly reduced to obtain the desired number of cells (∼10^9^ cells per production, 50–100 mL downstream processing volume). Increasing the harvesting cell density may be achieved by increasing seeding density and/or enhancing cell proliferation. However, depending on the culture system and the sensitivity of cell phenotype to the density change, seeding density may affect the secretion of autocrine and paracrine factors which have been shown to affect hPSC self‐renewal and differentiation [93]. To enhance cell proliferation, one could consider changing growth medium (containing growth factors) and the substrates which are usually coated with matrix proteins such as laminin, Matrigel, and fibronectin. However, any changes made in media and substrates need to be carefully compared.

### 5.2 Challenges in downstream processing for hPSC‐derived cardiomyocytes

For cardiomyocytes, the burden on the downstream bioprocessing is more severe than neural progenitors. The quantity of cells needed for therapeutic applications is higher for cardiomyocytes (∼10^10^ cells per production) than for neural progenitors (∼10^9^ per production) [5]. For all the steps in downstream bioprocessing of cardiomyocytes, it may require 10‐times more concentrated cell preparation or 10‐times more suspension volume compared with that of neural progenitors. Large‐scale purification of cardiomyocytes derived from human PSCs has been achieved using metabolic selection methods reaching 99% purity and yielding to the recovery of up to 2 × 10^9^ cells functional cells [18, 94, 95]. Cardiomyocytes also form aggregates more easily than neural progenitors and thus the yield of the depletion step could be lower. To efficiently label the cells with magnetic beads and separate different populations, cardiomyocytes need to be maintained as single cell suspension during downstream bioprocessing. Thus, the holding buffer and depletion buffer need to be formulated (e.g. anti‐clumping agents, biopolymers) to prevent cell clumping (Figure 1C). The scale of downstream processing of hPSC‐derived cardiomyocytes could be large given the quantity needed for therapeutic use. Therefore, scale‐up of different steps during downstream bioprocessing will be required (e.g. large‐scale centrifugation). Alternatively, an automation process designed for harvesting hPSC‐derived cardiomyocytes will be beneficial.

## 6 CONCLUSIONS AND PERSPECTIVES

Downstream bioprocessing has become the bottle neck in the production of hPSC‐derived cells for therapeutics after efficient differentiation of hPSCs is achieved. The identification of specific markers for desired lineages and the strategy to remove the undifferentiated cells are critical to produce safe cell therapy. Ideally, with high cell purity, depletion of unwanted cell types will reduce the burden on downstream bioprocessing. Developing a stringent quality control system and highly sensitive assays is required to ensure the consistent products after cell separation. The in vitro assays that can predict the in vivo effect are desirable to reduce the amount of preclinical studies. Applying the Quality by Design (QbD) strategy [96, 97] to understand the process and the product, better control on the process to produce consistent and safe cell products should be possible to fulfill the potential of hPSCs.

## CONFLICT OF INTEREST

The authors declare no conflict of interest.

## Data Availability

Data sharing not applicable – no new data generated.
